# Liver Fibrosis Biomarkers Accurately Exclude Advanced Fibrosis and Are Associated with Higher Cardiovascular Risk Scores in Patients with NAFLD or Viral Chronic Liver Disease

**DOI:** 10.3390/diagnostics11010098

**Published:** 2021-01-09

**Authors:** Stefano Ballestri, Alessandro Mantovani, Enrica Baldelli, Simonetta Lugari, Mauro Maurantonio, Fabio Nascimbeni, Alessandra Marrazzo, Dante Romagnoli, Giovanni Targher, Amedeo Lonardo

**Affiliations:** 1 Internal Medicine Unit, Pavullo Hospital, Azienda USL, 41026 Modena, Italy; a.marrazzo@ausl.mo.it; 2 Section of Endocrinology, Diabetes and Metabolism, Department of Medicine, University and Azienda Ospedaliera Universitaria Integrata of Verona, 37126 Verona, Italy; alessandro.mantovani@univr.it (A.M.); giovanni.targher@univr.it (G.T.); 3 Department of Biomedical, Metabolic and Neural Sciences, University of Modena and Reggio Emilia, 41126 Modena, Italy; enrica.baldelli@unimore.it; 4 Metabolic Medicine Unit, Ospedale Civile di Baggiovara, Azienda Ospedaliero-Universitaria, 41126 Modena, Italy; simonetta.lugari@libero.it (S.L.); maurantonio.mauro@aou.mo.it (M.M.); nascimbeni.fabio@aou.mo.it (F.N.); 5 Gastroenterology Unit, Ospedale Policlinico di Modena, Azienda Ospedaliero-Universitaria, 41126 Modena, Italy; romagnoli.dante@aou.mo.it; 6 Metabolic Syndrome Unit, Ospedale Civile di Baggiovara, Azienda Ospedaliero-Universitaria, 41126 Modena, Italy; lonardo.amedeo@aou.mo.it

**Keywords:** accuracy, aminotransferase, liver biopsy, liver enzymes, major cardiovascular events, mortality, NASH, fibrosis, non-invasive, sensitivity, specificity

## Abstract

Liver fibrosis predicts liver-related and cardiovascular outcomes in chronic liver disease patients. We compared the diagnostic performance of various liver fibrosis biomarkers for identifying histological significant/advanced fibrosis. Additionally, the correlations of such liver fibrosis biomarkers with cardiovascular risk (CVR) scores were evaluated. 173 patients with viral hepatitis (157 HCV and 16 HBV) and 107 with a non-alcoholic fatty liver disease (NAFLD) were consecutively enrolled. Various liver fibrosis biomarkers: aspartate aminotransferase (AST) to alanine aminotransferase (ALT) ratio (ARR), AST to Platelet Ratio Index (APRI), Fibrosis-4 (FiB-4), Forns index, NAFLD fibrosis score (NFS), BARD (body mass index (BMI), AAR, Diabetes) score, and Hepamet fibrosis score (HFS), were used to identify significant/advanced fibrosis. CVR was assessed by using the SCORE, the Progetto CUORE, or the Framingham risk scoring systems. Liver fibrosis biomarkers performed better in predicting advanced rather than significant liver fibrosis in all patients, regardless of chronic liver disease aetiology. Forns index and HFS performed best in predicting advanced fibrosis in patients with viral chronic liver disease and NAFLD. Lower cut-offs of these liver fibrosis biomarkers had high negative predictive values for advanced fibrosis overall, as well as in patients with NAFLD or viral chronic liver disease. FIB-4, Forns index, NFS, and HFS were positively correlated with SCORE and Framingham risk scores. In conclusion, liver fibrosis biomarkers accurately exclude advanced fibrosis and positively correlate with CVR scores in patients with chronic liver disease.

## 1. Introduction

Liver fibrosis defines the final result of repeated bouts of hepatocellular necrosis and liver inflammation, irrespective of the underlying aetiology of chronic liver disease [1]. Combined, metabolic and viral aetiologies account for a large proportion of fibrosing chronic liver disease globally [2]. The prototypic chronic liver disease of metabolic aetiology is Nonalcoholic Fatty Liver Disease (NAFLD). In many areas of the world NAFLD—which spans steatosis through non-alcoholic steatohepatitis (NASH) with/without fibrosis, cirrhosis, and hepatocellular carcinoma (HCC)—is the most common chronic liver disease [3] and its prevalence is escalating in parallel with the surge of diabesity [4,5]. Although the worldwide prevalence of viral chronic liver disease owing to infection with Hepatitis B (HBV) and C (HCV) viruses is declining thanks to effective preventive (HBV) and treatment strategies (HCV), viral chronic liver disease still continues to account for significant morbidity and mortality worldwide [6].

Liver fibrosis is a key determinant of the physiopathology and the natural course of both NAFLD and viral chronic liver disease, in as much as it progressively distorts normal hepatic architecture and, thereby, may eventually be conducive to hepatic insufficiency, portal hypertension, and HCC [1]. Further to these liver-related complications, the severity of liver fibrosis also dictates the risk of extra-hepatic cardio-metabolic complications [7,8,9,10,11,12,13]. Similar to NAFLD, HCV infection is increasingly identified as a systemic disease that may promote metabolic derangements and premature atherosclerosis [14,15].

On these grounds, the identification of subjects with significant/advanced fibrosis is key to implementing adequate treatment schedules and follow-up of patients with chronic liver disease [16,17,18]. Liver biopsy, which is the reference standard for the diagnosis and staging of hepatic fibrosis may cause patient discomfort and major complications [19,20]. Moreover, it may be exposed to sampling errors and diagnostic inaccuracies as a result of fibrosis being unevenly distributed throughout the liver tissue [18]. Therefore, liver biopsy should be reserved only for selected patients at risk of progressive liver disease [19,21]. The notion that they are clinically useful for helping in staging disease progression justifies the research of non-invasive biomarkers of liver fibrosis [19,21,22].

Widely available and inexpensive biomarkers of liver fibrosis include AST-to-ALT ratio (AAR), AST-to platelet ratio index (APRI), Fibrosis-4 (FIB-4), and Forns index, all of which have been applied in patients with chronic liver disease owing to different aetiologies [21,23]. Other non-invasive scores, such as NAFLD fibrosis score (NFS) and BARD (BMI, AAR, Diabetes), have specifically been generated for patients with NAFLD [24]. Generally speaking, these liver fibrosis biomarkers have shown better accuracy in excluding rather than in identifying significant/advanced fibrosis [21,23,25]. A new non-invasive fibrosis scoring system, namely the Hepamet fibrosis score (HFS), recently proposed by Ampuero et al. [26], has shown greater accuracy than the FIB-4 and NFS scores in identifying patients with NAFLD and advanced fibrosis.

In this complex scenario, the present study aims at comparing the diagnostic performance of various liver fibrosis biomarkers for identifying significant/advanced fibrosis on liver histology and their correlations with cardiovascular risk (CVR) scores in a sample of consecutive biopsied patients with either metabolic (NAFLD) or viral chronic liver disease.

## 2. Results

### 2.1. Characteristics of Study Cohort

The demographic, anthropometric, metabolic and histological characteristics of the study population are shown in Table 1. Overall, 66% were males, the mean age was 47.6 ± 11.5 years, and the mean BMI was 27.5 ± 4.5 Kg/m^2^. Among 280 patients with chronic liver disease, 107 had NAFLD (30 of whom had simple steatosis, and 77 had NASH), while 173 had viral hepatitis: 157 of whom had HCV (109 naïve and 48 treatment-experienced) and 16 had HBV (all treatment-naïve). None of the patients with viral chronic liver disease underwent previous treatment with direct acting anti-viral agents. One-hundred patients (36%) had significant fibrosis (42% NAFLD; 32% viral chronic liver disease) while thirty-eight patients (14%) had advanced fibrosis (13% NAFLD; 14% viral chronic liver disease) on liver biopsy. Considering NAFLD severity, the prevalence of significant fibrosis was 7% in simple steatosis and 56% in NASH while that of advanced fibrosis was 0% in simple steatosis and 18% in NASH. Histological fibrosis stages were distributed as follows: F0 33 (30.8%), F1 29 (27.1%), F2 31 (29.0%), F3 9 (8.4%), F4 5 (4.7%) according to Brunt/Kleiner et al. [27,28] in NAFLD (F0 21 (70.0%), F1 7 (23.3%), F2 2 (6.7%), F3 0 (0.0%), F4 0 (0.0%) in simple steatosis; F0 12 (15.6%), F1 22 (28.6%), F2 29 (37.6%), F3 9 (11.7%), F4 5 (6.5%) in NASH); F0 18 (10.4%), F1 57 (32.9%), F2 43 (24.9%), F3 31 (17.9%), F4 4 (2.3%), F5 15 (8.7%), F6 5 (2.9%) according to Ishak et al. [29] in viral chronic liver disease.

As reported in Table 1, when compared to those with viral chronic liver disease; patients with NAFLD were more likely to be overweight/obese, had a higher prevalence of diabetes and metabolic syndrome, as well as higher values of CVR scores (SCORE, Framingham risk score (FRS), Progetto CUORE), platelets, fasting glucose, fasting insulin, gamma-glutamyltransferase (GGT), total cholesterol, low-density lipoprotein (LDL)-cholesterol, triglycerides, serum uric acid, and ferritin. By contrast, these patients had lower values of aspartate aminotransferase (AST), alanine aminotransferase (ALT), γ-globulins, and high-density lipopoprotein (HDL)-cholesterol. Age, sex, prevalence of hypertension, fasting insulin, Homeostasis Model Assessment (HOMA)-estimated insulin resistance (IR), and albumin did not differ between the two groups of patients. Regarding histological features, as shown in Table 1, patients with NAFLD showed a greater prevalence of steatosis ≥5% than those with viral chronic liver disease. Significant/advanced fibrosis and prevalence of cirrhosis did not differ between the two groups. With regard to liver fibrosis biomarkers, patients with NAFLD had significantly lower values of AAR, APRI, FIB-4 score, and Forns when compared to those with viral chronic liver disease.

Clinical and laboratory characteristics of patients with and without significant/advanced fibrosis in the whole population and according to the aetiology of chronic liver disease are shown in Supplementary Tables S1–S4. Clinical and laboratory characteristics of NAFLD patients with and without NASH are shown in Supplementary Table S5.

### 2.2. Diagnostic Performance of Liver Fibrosis Biomarkers

**Figure 1:**
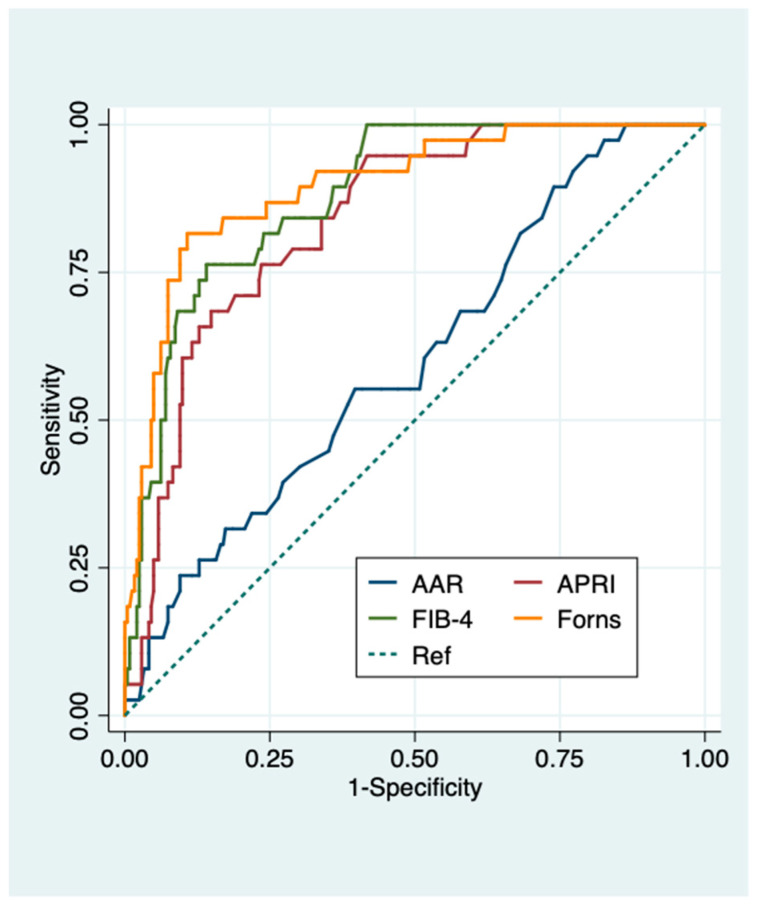
Diagnostic performance of serum biomarkers for predicting advanced fibrosis in all patients. Receiver operating characteristic (ROC) curves of serum biomarkers for the diagnosis of advanced fibrosis in all patients. AUROC (95% CI): 0.60 (0.51–0.70) for AAR, 0.84 (0.78–0.90) for APRI, 0.88 (0.83–0.93) for FIB-4, 0.90 (0.84–0.95) for Forns index. Advanced fibrosis was defined as histologic liver fibrosis ≥ F4 according to Ishak et al. [29] for viral chronic liver disease, and ≥F3 according to Brunt/Kleiner et al. for NAFLD [27,28]. AAR, AST to ALT ratio; APRI, AST to Platelet Ratio Index; Fib-4, fibrosis 4.

The diagnostic performance of liver fibrosis biomarkers (AAR, APRI, FIB-4, Forns) for predicting significant and advanced fibrosis was assessed in the whole population (Supplementary Table S6 and Table 2). Areas under the receiver operating characteristic (AUROCs) (95% confidence intervals (CI)) showed that liver fibrosis biomarkers were more accurate in predicting advanced rather than significant fibrosis (0.60 (0.51–0.70) vs 0.44 (0.40–0.51) for AAR; 0.84 (0.78–0.90) vs 0.68 (0.62–0.75) for APRI; 0.88 (0.83–0.93) vs 0.66 (0.59–0.73) for Fib-4; 0.90 (0.84–0.95) vs 0.69 (0.63–0.76) for Forns index) (Figure 1 and Supplementary Figure S1). FIB-4 using cut-off >3.25 and Forns index using cut-off <4.2 showed the best positive predictive value (PPV) (81.8%) and negative predictive value (NPV) (79.4%) for significant fibrosis, respectively. APRI, FIB-4 and Forns index using their lower cut-offs performed optimally to exclude (NPVs 98.4, 96.3 and 99.1%, respectively), rather than predict advanced fibrosis (PPVs 48.3, 63.6 and 75.7%, respectively) (Supplementary Table S6 and Table 2).

The diagnostic performance of liver fibrosis biomarkers for predicting significant (Supplementary Tables S7 and S8) and advanced fibrosis (Table 3 and Table 4) was then assessed in patients with chronic liver disease owing to different aetiologies, i.e., viral and NAFLD.

Forns index (0.77 (0.69–0.85)) and APRI (0.69 (0.59–0.80)) had the best AUROCs (95% CI) for predicting significant fibrosis in viral chronic liver disease and NAFLD patients, respectively (Supplementary Figures S2 and S3). AUROCs (95%CI) of all general liver fibrosis biomarkers (AAR, APRI, Fib-4, Forns index) showed a superior accuracy in predicting significant fibrosis among patients with viral chronic liver disease than among those with NAFLD (0.48 vs. 0.39 for AAR; 0.73 vs. 0.69 for APRI; 0.73 vs. 0.61 for FIB-4; 0.77 vs. 0.62 for Forns index). APRI and Forns index using their lower cut-offs showed the best NPV (84.8 and 89.3%) for significant fibrosis in viral chronic liver disease patients while all the scores performed poorly (NPVs < 70%) to exclude significant fibrosis in NAFLD patients (Supplementary Tables S7 and S8). FIB-4 and HFS using higher cut-offs had the best PPV for significant fibrosis in viral chronic liver disease and NAFLD (85.7 and 90.0%, respectively). Forns index showed the best combination of NPV (89.3%) and NPV (81.5%) for significant fibrosis in viral chronic liver disease patients (Supplementary Tables S7 and S8).

**Figure 2:**
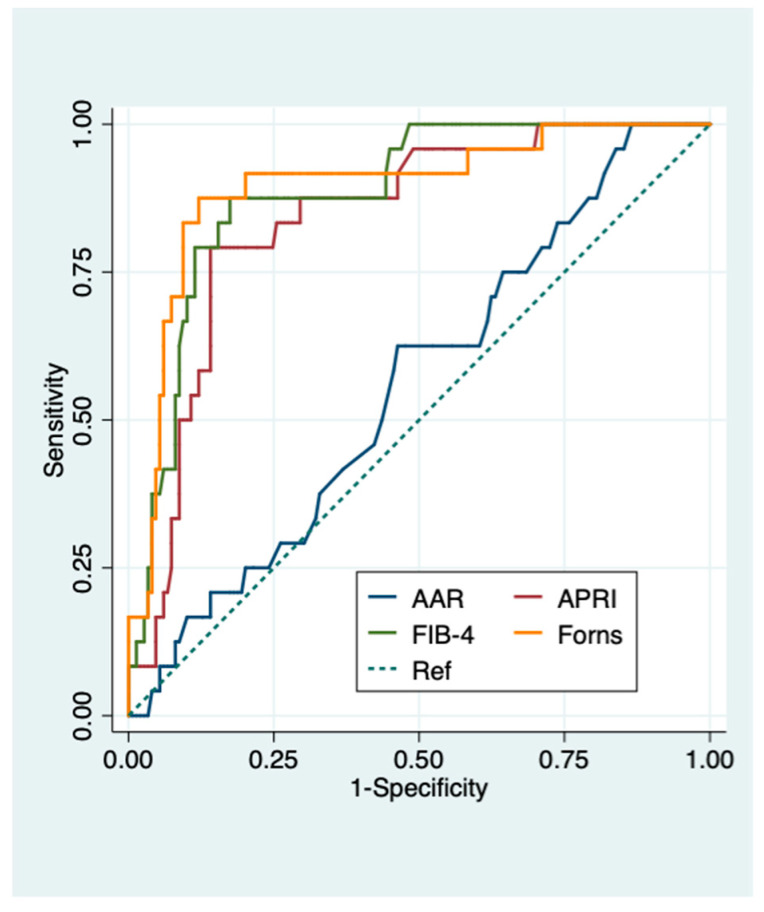
Diagnostic performance of serum biomarkers for predicting advanced fibrosis in viral chronic liver disease patients. ROC curves of serum biomarkers for the diagnosis of advanced fibrosis in viral chronic liver disease patients. AUROC (95% CI): 0.56 (0.44–0.68) for AAR, 0.84 (0.76–0.92) for APRI, 0.88 (0.82–0.95) for FIB-4, 0.89 (0.82–0.97) for Forns index. Advanced fibrosis was defined as histologic liver fibrosis ≥ F4 according to Ishak et al. [29]. AAR, AST to ALT ratio; APRI, AST to Platelet Ratio Index; Fib-4, fibrosis 4.

**Figure 3:**
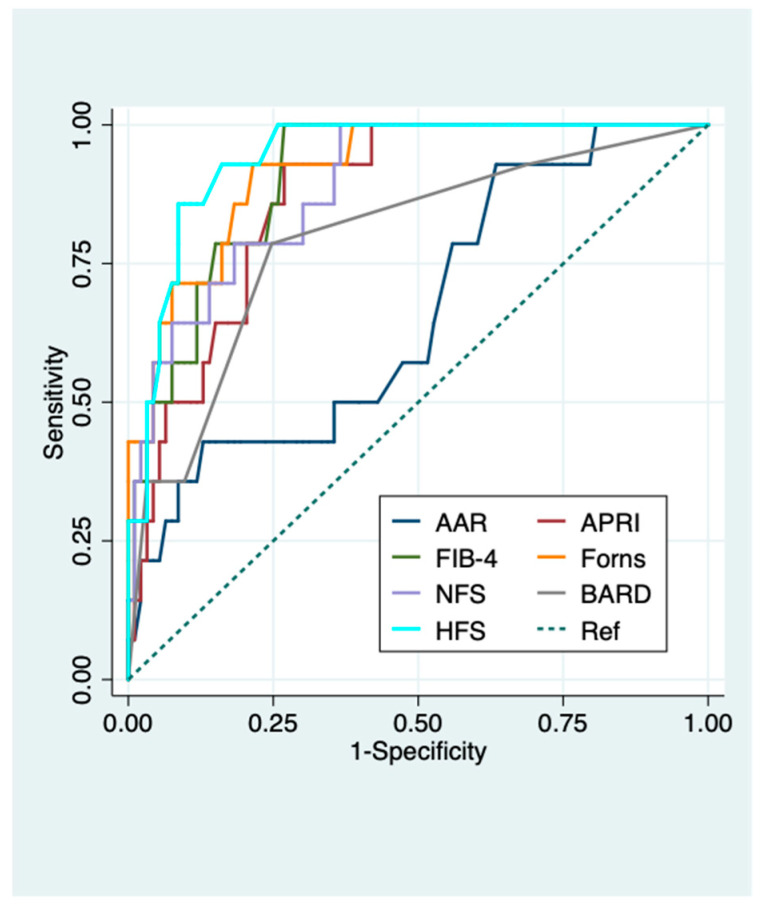
Diagnostic performance of serum biomarkers for predicting advanced fibrosis in NAFLD patients. ROC curves of serum biomarkers for the diagnosis of advanced fibrosis in NAFLD patients. AUROC (95% CI): 0.66 (0.50–0.82) for AAR, 0.87 (0.79–0.95) for APRI, 0.91 (0.84–0.97) for FIB-4, 0.92 (0.85–0.99) for Forns index, 0.89 (0.81–0.97) for NFS, 0.94 (0.90–0.99) for HFS, 0.79 (0.66–0.93) for BARD. Advanced fibrosis was defined as histologic liver fibrosis ≥F3 according to Brunt/Kleiner et al. for NAFLD [27,28]. AAR, AST to ALT ratio; APRI, AST to Platelet Ratio Index; BARD, BMI AST/ALT Ratio Diabetes; Fib-4, fibrosis 4; HFS, Hepamet fibrosis score; NFS, NAFLD fibrosis score.

Forns index (0.89(0.82–0.97)) and HFS (0.94 (0.90–0.99)) had the best AUROCs (95% CI) in predicting advanced fibrosis in viral chronic liver disease and NAFLD patients, respectively (Figure 2 and Figure 3).

AUROCs (95 CI%) of APRI, FIB-4 and Forns index all proved highly accurate in predicting advanced fibrosis with slightly better performance in patients with NAFLD compared to those with viral chronic liver disease (0.87 (0.79–0.95) vs. 0.84 (0.76–0.92) for APRI; 0.91 (0.84–0.97) vs. 0.88 (0.82–0.95) for FIB-4; 0.92 (0.85–0.99) vs. 0.89 (0.82–0.97) for Forns index). AAR performed poorly in predicting both significant and advanced fibrosis in the whole population and in patients with different chronic liver diseases (AUROCs (95%CI): 0.44 (0.40–0.51) and 0.60 (0.51–0.70) overall, 0.48 (0.38–0.57) and 0.56 (0.44–0.68) viral chronic liver disease, 0.39 (0.28–0.51) and 0.66 (0.50–0.82) NAFLD for significant and advanced fibrosis, respectively) (Supplementary Figures S1–S3 and Figure 1, Figure 2 and Figure 3). FIB-4, APRI, and Forns index using their lower cut-offs all showed excellent NPVs (95–100%) for advanced fibrosis in both NAFLD and viral chronic liver disease patients (Table 3 and Table 4). NFS, HFS, and BARD also had excellent NPVs (96–97%) for advanced fibrosis in NAFLD patients. Conversely, PPVs of all liver fibrosis biomarkers for advanced fibrosis were modest with FIB-4 showing the best PPV in both NAFLD and viral chronic liver disease patients (71.4% and 60%, respectively) (Table 3 and Table 4).

The finding that all liver fibrosis biomarkers, except for AAR, had NPVs greater than 95% for excluding advanced fibrosis using their lower cut-offs, implies that a significant proportion of liver biopsies ranging from 38% to 67% in our overall series, 32% to 74% in viral chronic liver disease and 48% to 80% in NAFLD patients could have been avoided with a negligible rate of false negatives (0–4%) using APRI, FIB-4, Forns, NFS, BARD, and HFS (Table 5).

### 2.3. Associations of Liver Fibrosis Biomarkers with Cardiovascular Risk Scoring Systems

Correlations between CVR scores (SCORE, Progetto CUORE, and FRS) and liver fibrosis biomarkers are shown in Table 6. All liver fibrosis biomarkers were significantly correlated with CVR scores, except for APRI with FRS, in the whole series, and with Progetto CUORE and FRS in patients with viral chronic liver disease. The best univariate correlations with SCORE were shown by FIB-4 and Forns index in the whole population (r = 0.462 and 0.468, respectively), as well as in patients with viral chronic liver disease (r = 0.476 and 0.454, respectively) and by NFS and Forns in those with NAFLD (r = 0.593 and 0.544, respectively). The best correlations with Progetto CUORE were shown by FIB-4 and Forns index in the whole population (r = 0.402 and 0.437, respectively), as well as in patients with viral chronic liver disease (r = 0.399 and 0.370, respectively) and by NFS and HFS in those with NAFLD (r = 0.658 and 0.643, respectively). FIB-4 and Forns index also had the best correlation with FRS in the whole population (r = 0.343 and 0.454, respectively) and in patients with viral chronic liver disease (r = 0.319 and 0.464, respectively). NFS and HFS showed the best correlation with FRS in NAFLD patients (r = 0.702 and 0.679, respectively). NFS showed the best correlation with all CVR in NAFLD patients. Supplementary Tables S1–S4 show that CVR scores tend to be higher among patients with either significant or advanced fibrosis compared to those individuals with either absent-to-mild or moderate histological fibrosis indirectly supporting the notion that liver fibrosis is associated with CVR.

## 3. Discussion

The main findings of our study, involving a large sample of 280 well-characterized patients with biopsy-confirmed chronic liver disease owing to viral and metabolic aetiology with a low prevalence of advanced liver fibrosis, are as follows: (a) liver fibrosis biomarkers accurately exclude advanced fibrosis in both metabolic and viral chronic liver disease; (b) liver fibrosis biomarkers are well correlated with CVR scores assessing 10-year fatal and non-fatal cardiovascular disease (CVD) events.

### 3.1. Liver Fibrosis Biomarkers Accurately Exclude Advanced Fibrosis in Metabolic and Viral Chronic Liver Disease

In this study, liver fibrosis biomarkers performed better in excluding advanced fibrosis (assessed with NPVs) than previously reported in both NAFLD [30] and viral chronic liver disease patients [31,32,33]. Our data have shown that, overall, NPVs range 96–99%, 96–100% in NAFLD, and 97–98% in viral chronic liver disease compared to previous reported NPVs ranging 89–93% in NAFLD [30], and 73–95% in viral chronic liver disease [31,32,33]. In our study, the Forns index showed the highest NPV in excluding advanced fibrosis in patients with viral chronic liver disease (NPV 98%, as well as APRI) and also in those with NAFLD (NPV 100%) where it was first applied. Moreover, the present investigation, the first external validation of the new HFS, shows that HFS, compared to other liver fibrosis biomarkers, has good accuracy in excluding advanced fibrosis (NPV 96.5%) and the best diagnostic accuracy (AUROC 0.94) to differentiate F3–F4 from F0–F2 in NAFLD patients, in line with the results of Ampuero et al. [26]. Conversely, the Forns index showed the best diagnostic performance in predicting advanced fibrosis in patients with viral chronic liver disease (AUROC 0.89). Nevertheless, all liver fibrosis biomarkers in our study showed sub-optimal PPVs (≤76%) for detecting advanced fibrosis, such as reported by others [16,26,30,33,34,35,36,37,38] indicating that their clinical utility lies in ruling out rather than confirming advanced fibrosis. Recent studies suggest that “FAST” score, i.e., a combination of various indices obtained with Fibroscan (LSM, CAP), and AST may work excellently to this end [39].

Our study confirms that, among the various liver fibrosis biomarkers, HFS demonstrates the best diagnostic performance. This may be explained by the fact that HFS was originally developed on a large multi-centre international cohort [26]; its algorithm—at variance with other scores—is already adjusted for confounding variables such as older age (≥65 years) [40]; and in subjects without diabetes, it includes the calculation of HOMA-IR, a reliable marker of insulin resistance which is intimately associated with the development and progression of the whole NAFLD spectrum, including advanced fibrosis [41]. Data on the diagnostic accuracy of the Forns index in NAFLD patients are scant and limited to the prediction of significant fibrosis [34]. The Forns index is calculated based on cholesterol serum levels, which are negatively associated with the stage of liver fibrosis and may predict the diagnosis of NASH [41,42,43].

### 3.2. Clinical Implications of Findings: Proportion of Spared Biopsies and NAFLD Screening

Our findings support the notion that liver fibrosis biomarkers may be adopted in clinical practice to accurately exclude advanced liver fibrosis. This would allow avoiding a consistent number of liver biopsies: 40–60% in the whole population of patients using APRI, FIB-4 and Forns index lower cut-offs; 30–70% in those with the viral chronic liver disease using APRI, FIB-4, and Forns lower cut-offs and also APRI intermediate cut-offs; 50–80% in those with NAFLD using FIB-4, Forns index, NFS, BARD, and HFS lower cut-offs. In all groups, the false negative rate was very low (0–4%). Our results outperform those previously reported by McPherson et al. in a European NAFLD patient population featuring a 19% prevalence of advanced fibrosis. McPherson showed that 40–70% of biopsies were avoidable; with a 5–8% false negative rate [44]. Similarly, Treeprasertsuk et al. in Asian patients with NAFLD featuring a 6% prevalence of advanced fibrosis found that the proportion of potentially avoidable liver biopsies was 40–80% with 1–4% false negatives [45]. At variance with other studies, generally reporting a prevalence of advanced fibrosis ranging around 20% to 30% [26,33,35,37,44,46,47], our patient population was characterized by a lower prevalence of advanced fibrosis (14%) suggesting a more liberal use of liver biopsy during the years when our study was conducted.

Our results support the recommendations of current guidelines which suggest screening NAFLD patients with liver fibrosis biomarkers in order to identify those with either suspected advanced fibrosis or indeterminate values. It is these patients who should undergo further non-invasive assessments of liver fibrosis with sono-elastographic techniques [21,48,49] or liver biopsy—the only diagnostic modality remaining to accurately diagnose and stage the severity of NASH [25].

Research on diagnostic tools for non-invasive and accurate identification of NASH and severity of fibrosis is actively progressing and magnetic resonance elastography is the most promising; however, this imaging technique is very expensive and of limited availability [16,21] particularly, but not only, in developing countries. Another emerging technique is the semiquantitative/quantitative liver ultrasonography, which may be combined with other non-invasive tools, such as liver fibrosis biomarkers and sono-elastography [50,51].

### 3.3. Liver Fibrosis Biomarkers are Correlated with CVR Scores

A consistent body of literature supports the notion that the liver is a key modulator of CVR and that such a risk is associated with the histological features of liver disease both in NAFLD and in HCV infection [10,52,53,54,55,56,57,58]. Based on this robust rationale, our study is the first to report the correlation between the widely used liver fibrosis biomarkers and CVR scores, including the Progetto CUORE, which has specifically been developed for the Italian general population. Moderate liver fibrosis, although to a lesser extent than advanced fibrosis, has also been associated with increased mortality in patients with NAFLD but we cannot accurately rule this out with available non-invasive tests. Therefore, it has been suggested that patients without advanced fibrosis should actively be followed with a focus on metabolic co-morbidities in order to reduce CVR and should be retested after three years to identify those with disease progression [59].

Longitudinal studies have found that liver fibrosis biomarkers may predict the risk of mortality and CVD events in NAFLD [60,61,62,63,64]. Data in viral chronic liver disease, whose epidemiology and natural history has been altered by highly effective, direct-acting antiviral therapies, are eagerly awaited. Conversely, NAFLD is an epidemiologically growing chronic liver disease for which no effective pharmacological treatment has been licensed so far. The NAFLD/NASH pandemic is leading to an increase in CVD, underlining the need to develop non-invasive tools able to assess the progression of liver disease and stratify CVR [12,65,66]. In our study, FIB-4 and NFS scores showed a moderate to strong correlation with FRS in NAFLD patients, which was higher than previously reported [67,68,69]. We are the first to report a correlation between the newly proposed HFS index and CVR scores as well as between different liver fibrosis biomarkers and the SCORE equation estimating 10-year fatal CVD risk in European populations. FIB-4 and Forns index proved moderately correlated with CVR scores in patients with viral chronic liver disease. NFS, followed by HFS, exhibited a moderate to strong correlation with CVR scores in NAFLD patients. At variance with a recent study [69], we found a correlation between liver fibrosis biomarkers and CVR assessed with the Italian scoring system “Progetto CUORE”. This is important given that locally validated scoring systems must be used to obtain an accurate assessment of CVR [70].

### 3.4. Clinical Implications of Findings: May Liver Fibrosis Biomarkers Be Used as an Adjunct to Traditional CVR Scoring Systems?

Our study indicates that higher scores of estimated CVR are found among those with more advanced liver fibrosis (Table 6; Supplementary Tables S1–S4). This association of biomarkers of liver fibrosis with scores obtained with conventional scoring systems raises the possibility to directly gauge CVR through the use of biomarkers of liver fibrosis in clinical practice. Although this notion has a robust rationale such as discussed above, we believe that, for the time being, this research question should best be addressed with appropriate follow-up studies evaluating the occurrence of major cardiovascular events in chronic liver disease patients owing to either metabolic or viral aetiology and followed-up for adequate periods of time. These studies are eagerly awaited given the need to perform an innovative, non-invasive joint (“one-shot”) assessment of both cardio-metabolic and hepatological risks through non-invasive markers of liver fibrosis. Additionally, we point out that the series of patients labelled in the present study as “viral” mainly consists of individuals with chronic hepatitis C, while those with chronic hepatitis B are only a minority (Table 1). This is important to say, given that HCV and HBV may have a different and potentially opposite impact on the development of hepatic steatosis and cardio-metabolic risk [71,72].

### 3.5. Strengths and Limitations

The strengths of this study include the large sample of well-characterized patients with biopsy-proven chronic liver disease owing to different aetiologies, which represents the first external validation of HFS. Conversely, the cross-sectional design of the study limits our ability to determine the causality and temporality of the observed associations. Moreover, the mono-centric nature of our study, which was performed at a tertiary liver centre is another limitation of our research. Nevertheless, the proportion of advanced fibrosis in our study was lower than that of other similar studies, which renders our study population more similar to an unselected, general practice setting. Recent studies evaluating the diagnostic performance of liver fibrosis biomarkers for detecting advanced fibrosis in NAFLD patients according to the presence of type 2 diabetes have yielded conflicting results [73,74]. Unfortunately, the proportion of individuals with type 2 diabetes in our series did not allow us to perform separate analyses in patients with and without type 2 diabetes. Clearly, our findings remain to be validated in a prospective confirmation cohort as well as in non-Caucasian patients.

Our study was aimed at describing the performance of non-invasive biomarkers in detecting hepatic fibrosis in a series of patients with chronic liver disease owing to either metabolic or viral aetiology. Even in the direct-acting antivirals era, a proportion of underprivileged patients with HCV infection, usually owing to a limited access to health resources, will follow the natural course of disease [75]. In others, chronic liver disease owing to HCV infection is at an advanced stage of fibrosis when they are first submitted to direct-acting antivirals [76]. Moreover, direct-acting antivirals are expensive and poorly available in many areas of the world [77] and a small proportion of treated patients will fail to clear viral infection [78]. Finally, we believe that the association of liver fibrosis biomarkers with CVR scores fully maintains its proof-of-concept significance even in the direct-acting antivirals era.

## 4. Materials and Methods

Consecutive patients submitted to liver biopsy at a referral Liver Clinic at the University Hospital of Modena, Italy, between the years 2001 and 2012, with a biopsy-proven diagnosis of either NAFLD or viral (HCV or HBV-related) chronic liver disease were enrolled in a retrospective study. The liver biopsy was performed as part of the diagnostic work-up of abnormal liver tests, suspected liver diseases, or grading/staging of known chronic liver disease. Criteria for exclusion from the study were as follows: (a) diagnosis of alcoholic liver disease or other liver diseases (autoimmune, heredo-metabolic) based on clinical data/appropriate testing and histological criteria; (b) incomplete data to calculate all the non-invasive liver fibrosis biomarkers; (c) the presence of decompensated cirrhosis; (d) either primary or metastatic liver cancer; a history of major cardiovascular events.

The liver biopsies were performed, all subjects gave their informed consent for inclusion before they participated in the study. The study was conducted in accordance with the Declaration of Helsinki, and the protocol was approved by the Ethics Committee of Modena (Project identification code 0035241/20). All enrolled patients were interviewed regarding their familial and personal medical history, notably including daily alcohol consumption. All patients underwent complete physical examination, assessment of anthropometric indices, recording of blood pressure, and routine blood sampling for laboratory tests. Blood test results were performed either on the day of liver biopsy or within one month. HOMA-IR, body mass index (BMI), waist circumference, impaired fasting glycemia (IFG), diabetes, hypertension, and Metabolic Syndrome were defined based on standard criteria [79,80,81,82]. CVR was assessed based on widely adopted scores validated in European and American populations: the SCORE estimating the total 10-year risk for fatal CVD, the Progetto CUORE, and FRS predicting the total 10-year risk for CVD [83,84,85]. The “Progetto CUORE” CVR scoring system has been specifically developed and validated for the Italian general population [84]. This is important given that commonly used scoring systems have been developed and validated in English-speaking countries and may overestimate true CVR in Southern European populations [86]. The diagnosis of NAFLD was based on ultrasonographic/biopsy-proven fatty liver in the absence of excessive alcohol consumption (defined as daily alcohol intake >20 g) and other competing aetiologies of liver disease [87].

The diagnosis of HCV infection was confirmed by polymerase chain reaction testing in HCV-Ab positive patients, and after excluding competing aetiologies of liver disease [88]. The diagnosis of HBV infection was based on a typical serological pattern determined by a standard commercially available enzyme-linked immunosorbent assay [89].

### 4.1. Histological Evaluation

Liver biopsies were evaluated by a single experienced liver pathologist (L.L.); only biopsy samples at least 15 mm long with at least 10 portal tracts were considered eligible for analysis.

Biopsy specimens of patients with NAFLD were scored according to the Brunt criteria [27]. Fibrosis was staged (F0 = none; F1 = perisinusoidal/pericellular or portal/periportal; F2 = perisinusoidal/pericellular plus portal/periportal; F3 = bridging; F4 = cirrhosis) according to the Brunt and Kleiner criteria [27,28]. Biopsy specimens of patients with chronic viral hepatitis (HCV and HBV) were scored according to Ishak et al. [29]. Fibrosis was staged as follows: F0 = none; F1 = fibrous expansion of some portal areas with or without short fibrous septa; F2 = fibrous expansion of most portal areas with or without short fibrous septa; F3 = fibrous expansion of most portal areas with an occasional portal to portal bridging; F4 = fibrous expansion of portal areas with marked portal-portal and portal-central bridging; F5 = marked bridging with occasional nodules (incomplete cirrhosis); F6 = cirrhosis, probable or definite [29].

Liver fibrosis was considered: (a) significant in the presence of fibrosis stage ≥ F2 for NAFLD patients and ≥F3 for those with viral chronic liver disease; and (b) advanced (i.e., bridging fibrosis) in the presence of fibrosis stage ≥F3 for NAFLD patients and ≥F4 for those with viral chronic liver disease [30,34,35].

### 4.2. Liver Fibrosis Biomarkers

The general (AST/ALT, APRI, Fib-4 and Forns) and NAFLD-specific (NFS, BARD and Hepamet) non-invasive liver fibrosis biomarkers were determined according to published formulas. AAR was calculated as: AST(IU/l)/ALT(IU/l) [90]. APRI score was calculated as: AST (IU/l)/platelet count (× 10^9^/l) × 100 [91]; FIB-4 score as: age × AST (IU/l)/platelet count (×10^9^/l) × √ALT (IU/l) [43]; Forns index as: 7.811–3.131 ln (platelet count (×10^9^/l)) + 0.781 ln (GGT (IU/L)) + 3.467 ln (age (years))–0.014 (cholesterol (mg/dl)) [92]; NFS as: –1.675 + 0.037 × age (years) + 0.094 × BMI (kg/m^2^) + 1.13 × IFG or diabetes (yes = 1, no = 0) + 0.99 × AST/ALT ratio – 0.013 × platelet (×10^9^/l) – 0.66 × albumin (g/dl) [47]; BARD score (ranging 0–4) as: BMI (>28 = 1; <28 = 0) + AST/ALT ratio (>0.8 = 2; <0.8 = 0) + diabetes (yes = 1, no = 0) [93]; HFS as: 1/(1 + e ^(5.390–0.986 (if Age 45–64 years) – 1.719 (if Age ≥ 65 years) + 0.875 (if Male sex) – 0.896 (if AST 35–69 IU/L) – 2.126 (if AST ≥ 70 IU/L) – 0.027 (if Albumin 4–4.49 g/dL) – 0.897 (if Albumin <4 g/dL) – 0.899 (if HOMA 2–3.99 with no Diabetes Mellitus) –1.497 (if HOMA ≥ 4 with no Diabetes Mellitus) – 2.184 (if Diabetes Mellitus) – 0.882 (if Platelets 155–219 × 1000/µL) – 2.233 (if Platelets < 155 × 1000/µL))^) [26].

Cut-offs for predicting significant and advanced liver fibrosis were applied based on the available literature, including original reports, validation studies and meta-analytic reviews [30,34,35,36,44,46,47,90,91,92,93]. The cut-offs adopted in the above cited studies were as follows: ≤0.5 for APRI, <1.45 (NAFLD/viral chronic liver disease) or <1.3 (NAFLD) for Fib-4, <4.2 for Forns index, <−1.455 for NFS, <0.12 for HFS to exclude significant/advanced fibrosis; >0.7, >1.0, >1.5 for APRI, >3.25 (NAFLD/viral chronic liver disease) or >2.67 (NAFLD) for Fib-4, >6.9 for Forns index, >0.675 for NFS, >0.47 for HFS to predict significant/advanced fibrosis.

### 4.3. Statistical Analysis

The Kolmogorov–Smirnov test was used to assess the normality of variables. The results are shown as means ± SD for continuous variables normally distributed and medians (25–75th percentile) for variables not normally distributed. Categorical variables are shown as relative or absolute proportions. Comparisons between the means of continuous variables were performed using the unpaired 2-tailed Student’s t-test for normally distributed variables, whereas the Mann–Whitney test was performed for non-normally distributed or ordinal variables. Chi-square test or Fisher’s exact test were used to compare nominal variables.

The diagnostic performance of different liver fibrosis biomarkers for predicting significant and advanced fibrosis according to the above-reported cut-offs was evaluated by calculating the sensitivity, specificity, PPV, NPV, accuracy, positive and negative likelihood ratio with standard formulas. Moreover, the AUROC curves and their corresponding 95% CI were also calculated. Spearman’s rho (ρ) or Pearson’s r were used where appropriate for the analysis of correlations between liver fibrosis biomarkers and CVR scoring systems (SCORE, Progetto CUORE, and Framingham risk scores).

A *p*-value <0.05 was considered to be statistically significant. Statistical analyses were performed by using the statistical software package SPSS, version 24.0 for Windows (SPSS Inc., Chicago, Illinois, USA) and STATA Stata version 14 for Windows (StataCorp, College Station, Texas).

## 5. Conclusions

We have shown that liver fibrosis biomarkers may accurately exclude (rather than confirm) advanced liver fibrosis in patients with NAFLD or viral chronic liver disease and correlate with CVD risk scores in a population with a low prevalence of advanced fibrosis. The new HFS had the best diagnostic performance for diagnosing advanced fibrosis among NAFLD patients.

The combination of various non-invasive tools (liver fibrosis biomarkers, sono-elastographic techniques and, if available, magnetic resonance elastography) may allow an accurate and “one-shot” simultaneous non-invasive assessment/stratification of the risks of both liver fibrosis and cardio-metabolic events in patients with chronic liver disease owing to the either metabolic or viral origin. This hypothesis deserves specific investigation through appropriate follow-up studies of well-characterized cohorts of patients with chronic liver disease.

## Figures and Tables

**Table 1:** Characteristics of the study population.

	All (*n* = 280)	Viral CLD * (*n* = 173)	NAFLD (*n* = 107)	*p* ^†^
***Biometrics***				
Age (years)	47.6 ± 11.5	47.7 ± 11.3	47.6 ± 11.9	0.983
Male/Female ratio (*n*, %)	185/95 (66/34)	108/65 (62/38)	77/30 (72/28)	0.119
BMI (kg/m^2^)	27.5 ± 4.5	26.0 ± 3.9	29.5 ± 4.5	<0.001
Waist Circumference (cm)	97.1 ± 12.3	91.0 ± 9.9	101.8 ± 11.9	<0.001
Hypertension (*n*, %)	82 (29)	44 (26)	38 (36)	0.081
Type 2 diabetes (*n*, %)	46 (16)	13 (8)	33 (31)	<0.001
Metabolic syndrome (*n*, %)	77 (28)	21 (12)	56 (52)	<0.001
***CVR scores***				
SCORE	0.5 (0.1 ÷ 1.4)	0.4 (0.1 ÷ 1.4)	0.7 (0.2 ÷ 1.7)	0.052
FRS	7.3 (3.6 ÷ 14.8)	6.7 (3.6 ÷ 13.4)	8.7 (4.0 ÷ 19.9)	0.026
Progetto CUORE	1.9 (0.8 ÷ 4.8)	1.5 (0.6 ÷ 4.1)	2.5 (1.1 ÷ 6.0)	0.002
***Laboratory***				
Platelet count (×10^3^/mm^3^)	212.6 ± 61.4	206.2 ± 61.3	223.0 ± 60.2	0.025
Fasting glucose (mg/dL)	95.0 (87.0 ÷ 103.0)	93.5 (85.0 ÷ 100.0)	98.0 (91.0 ÷ 116.0)	<0.001
Fasting insulin (mIU/L)	10.7 (7.2 ÷ 15.8)	9.6 (7.1 ÷ 15.7)	11.5 (8.0 ÷ 15.9)	0.244
HOMA-IR score	2.6 (1.6 ÷ 4.0)	2.4 (1.5 ÷ 3.8)	2.9 (1.9 ÷ 4.3)	0.148
AST (U/L)	39.7 (28.0 ÷ 58.9)	43.0 (32.0 ÷ 69.7)	33.0 (26.0 ÷ 49.0)	<0.001
ALT (U/L)	41.5 (64.5 ÷ 119.7)	71.0 (44.0 ÷ 130.2)	58.0 (40.0 ÷ 91.0)	0.019
GGT (U/L)	41.0 (27.4 ÷ 76.8)	40.0 (24.0 ÷ 62.1)	49.0 (33.0 ÷ 109.0)	0.003
Albumin (g/dL)	4.4 ± 0.5	4.4 ± 0.4	4.5 ± 0.5	0.052
γ-globulin (mg/dL)	1.3 ± 0.4	1.4 ± 0.4	1.1 ± 0.3	<0.001
Total Cholesterol (mg/dL)	186.7 ± 44.8	171.8 ± 37.6	210.8 ± 45.1	<0.001
HDL-Cholesterol (mg/dL)	46.4 ± 13.9	48.3 ± 14.2	44.4 ± 13.5	0.049
LDL-Cholesterol (mg/dL)	118.9 ± 39.5	102.3 ± 32.5	135.2 ± 39.1	<0.001
Triglycerides (mg/dL)	69.0 (97.0 ÷ 147.0)	82.0 (62.0 ÷ 113.0)	146.0 (92.0 ÷ 228.0)	<0.001
Serum uric acid (mg/dL)	5.3 ± 1.4	4.8 ± 1.2	6.0 ± 1.5	<0.001
Ferritin (mg/dL)	150.0 (79.0 ÷ 252.5)	134.0(64.0 ÷ 252.5)	178.0 (98.5 ÷ 258.3)	0.036
***Liver histology***				
Steatosis ≥ 5% (*n*, %)	184 (66)	75 (43)	107 (100)	<0.001
Significant fibrosis (*n*, %)	100 (36)	55 (32)	45 (42)	0.095
Advanced fibrosis (*n*, %)	38 (14)	24 (14)	14 (13)	1.000
Cirrhosis (*n*, %)	10 (4)	5 (3)	5 (5)	0.513
***Liver fibrosis biomarkers***				
AAR	0.62 (0.49 ÷ 0.73)	0.64 (0.52 ÷ 0.74)	0.56 (0.46 ÷ 0.69)	0.002
APRI	0.57 (0.35 ÷ 0.88)	0.64 (0.40 ÷ 1.08)	0.43 (0.30 ÷ 0.69)	<0.001
FIB-4	1.05 (0.72 ÷ 1.65)	1.21 (0.82 ÷ 1.92)	0.84 (0.63 ÷ 1.29)	<0.001
Forns	4.9 ± 1.8	5.1 ± 1.8	4.5 ± 1.8	0.019

* HCV: *n* = 157; HBV: *n* = 16. ^†^ NAFLD vs. viral CLD. Data are expressed as means (±SD) for continuous variables normally distributed or as medians (25–75th percentile) for those not normally distributed, and as frequencies (percentages) for categorical variables. AAR, AST to ALT ratio; ALT, alanine aminotransferase; APRI, AST to Platelet Ratio Index; AST, aspartate aminotransferase; BMI, Body mass index; CLD, chronic liver disease; CVR, cardiovascular risk; FIB-4, fibrosis 4; FRS, Framingham risk score; GGT, gamma-glutamyltransferase; HBV, hepatitis B virus; HCV, hepatitis C virus; HDL-C, high-density lipoprotein; HOMA-IR, homeostasis model assessment of insulin resistance; LDL, low-density lipoprotein; NAFLD, non-alcoholic fatty liver disease.

**Table 2:** Diagnostic performance of liver fibrosis biomarkers for predicting advanced liver fibrosis in the whole population (*n* = 280).

	Cut-Offs	SE	SP	PPV	NPV	ACC	LR+	LR−	AUROC (95% CI)
**AAR**	≥0.8	26.3	84.3	20.8	87.9	76.4	1.68	0.87	0.60 (0.51–0.70)
	>1	13.2	93.8	25.0	87.3	82.9	2.12	0.93	
**APRI**	>0.5	94.7	49.2	22.6	98.4	55.4	1.86	0.11	0.84 (0.78–0.90)
	>1	63.2	87.2	43.6	93.8	83.9	4.93	0.42	
	>1.5	36.8	93.8	48.3	90.4	86.1	5.94	0.67	
**FIB-4**	≥1.45	81.6	74.4	33.3	96.3	75.4	3.18	0.25	0.88 (0.83–0.93)
	>3.25	36.8	96.7	63.6	90.7	88.6	11.15	0.65	
**Forns**	≥4.2	97.4	43.8	21.4	99.1	51.1	1.73	0.06	0.90 (0.84–0.95)
	>6.9	40.6	95.7	75.7	83.1	82.1	9.51	0.62	

Advanced fibrosis was defined as histologic liver fibrosis ≥ F4 according to Ishak et al. [29] for viral chronic liver disease, and ≥F3 according to Brunt/Kleiner et al. for NAFLD [27,28]. AAR, AST to ALT ratio; ACC, accuracy; ALT, alanine aminotransferase; APRI, AST to Platelet Ratio Index; AST, aspartate aminotransferase; AUROC, area under the receiver operating characteristics; BLF, biomarkers of liver fibrosis; Fib-4, fibrosis 4; LR, likelihood ratio; NPV, negative predictive value; PPV, positive predictive value; SE, sensitivity; SP, specificity.

**Table 3:** Diagnostic performance of liver fibrosis biomarkers for predicting advanced fibrosis in patients with viral chronic liver disease (*n* = 173).

	Cut-Offs	SE	SP	PPV	NPV	ACC	LR+	LR−	AUROC (95% CI)
**AAR**	≥0.8	20.8	81.9	15.6	86.5	73.4	1.15	0.97	0.56 (0.44–0.68)
	>1	8.3	92.6	15.4	86.3	80.9	1.13	0.99	
**APRI**	>0.5	95.8	38.9	20.2	98.3	46.8	1.57	0.11	0.84 (0.76–0.92)
	>1	79.2	82.6	42.2	96.1	82.1	4.54	0.25	
	>1.5	50.0	90.6	46.2	91.8	85.0	5.32	0.55	
**FIB-4**	≥1.45	87.5	67.1	30.0	97.1	69.9	2.66	0.19	0.88 (0.82–0.95)
	>3.25	37.5	96.0	60.0	90.5	87.9	9.31	0.65	
**Forns**	≥4.2	95.8	36.9	19.7	98.2	45.1	1.52	0.11	0.89 (0.82–0.97)
	>6.9	66.7	92.6	59.3	94.5	89.0	9.03	0.36	

Advanced fibrosis was defined as histologic liver fibrosis ≥ F4 according to Ishak et al. [29]. AAR, AST to ALT ratio; ACC, accuracy; ALT, alanine aminotransferase; APRI, AST to Platelet Ratio Index; AST, aspartate aminotransferase; AUROC, area under the receiver operating characteristics; BLF, biomarkers of liver fibrosis; CI, confidence intervals; FIB-4, fibrosis 4; LR, likelihood ratio; NPV, negative predictive value; PPV, positive predictive value; SE, sensitivity; SP, specificity.

**Table 4:** Diagnostic performance of liver fibrosis biomarkers for predicting advanced fibrosis in patients with NAFLD (*n* = 107).

	Cut-Offs	SE	SP	PPV	NPV	ACC	LR+	LR−	AUROC (95% CI)
**AAR**	≥0.8	35.7	88.2	31.3	90.1	81.3	3.02	0.73	0.66 (0.50–0.82)
	>1	21.4	95.7	42.9	89.0	86.0	4.98	0.82	
**APRI**	>0.5	92.9	65.6	28.9	98.4	69.2	2.70	0.11	0.87 (0.79–0.95)
	>1	35.7	94.6	50.0	90.7	86.9	6.64	0.68	
	>1.5	14.3	98.9	66.7	88.5	87.9	13.29	0.87	
**Fib-4**	≥1.3	78.6	83.9	42.3	96.3	83.2	4.87	0.26	0.91 (0.84–0.97)
	≥1.45	71.4	86.0	43.5	95.2	84.1	5.11	0.33	
	>2.67	35.7	96.8	62.5	90.9	88.8	11.07	0.66	
	>3.25	35.7	97.9	71.4	91.0	89.7	16.61	0.66	
**Forns**	≥4.2	100.0	54.8	25.0	100.0	60.8	2.21	0.00	0.92 (0.85–0.99)
	>6.9	42.9	95.7	60.0	91.8	88.8	9.96	0.60	
**NFS**	≥−1.455	85.7	64.5	26.7	96.8	67.3	2.42	0.22	0.89 (0.81–0.97)
	>0.675	42.9	96.8	66.7	91.8	89.7	13.29	0.59	
**BARD**	≥2	78.6	75.3	32.4	95.9	75.7	3.18	0.29	0.79 (0.66–0.93)
**HFS**	≥0.12	78.6	89.3	52.4	96.5	87.9	7.31	0.24	0.94 (0.90–0.99)
	>0.47	50.0	96.8	70.0	92.8	90.7	15.5	0.52	

Advanced fibrosis was defined as histologic liver fibrosis ≥ F3 according to Brunt/Kleiner et al. [27,28]. Abbreviations: AAR, AST to ALT ratio; ACC, accuracy; ALT, alanine aminotransferase; APRI, AST to Platelet Ratio Index; AST, aspartate aminotransferase; AUROC, area under the receiver operating characteristics; BARD, BMI AST/ALT Ratio Diabetes; BLF, biomarkers of liver fibrosis; CI, confidence intervals; FIB-4, fibrosis 4; HFS, Hepamet fibrosis score; LR, likelihood ratio; NFS, NAFLD fibrosis score; NPV, negative predictive value; PPV, positive predictive value; SE, sensitivity; SP, specificity.

**Table 5:** Proportion of chronic liver disease patients without advanced fibrosis by liver fibrosis biomarkers who could have potentially avoided liver biopsy.

		All		Viral CLD		NAFLD	
	Cut-Offs	Patients, *n* (%)	FN, *n* (%)	Patients, *n* (%)	FN, *n* (%)	Patients, *n* (%)	FN, *n* (%)
**AAR**	<0.8	232/280 (83)	28 (12)	141/173 (82)	19 (14)	91/107 (85)	9 (10)
	≤1	260/280 (93)	33 (13)	160/173 (93)	22 (14)	100/107 (94)	11 (11)
**APRI**	<0.5	121/280 (52)	2 (2)	59/173 (34)	1 (2)	62/107 (58)	1 (2)
	<0.7	177/280 (63)	8 (5)	93/173 (54)	3 (3)	84/107 (79)	5 (6)
	<1	225/280 (80)	14 (6)	128/173 (74)	5 (4)	97/107 (91)	9 (9)
**FIB-4**	<1.3	173/280 (62)	6 (4)	92/173 (53)	3 (3)	81/107 (76)	3 (4)
	<1.45	187/280 (67)	7 (4)	103/173 (60)	3 (3)	84/107 (79)	4 (5)
**Forns**	<4.2	107/280 (38)	1 (1)	56/173 (32)	1 (2)	51/107 (48)	0 (0)
**NFS**	<−1.455	_	_	_	_	62/107 (58)	2 (3)
**BARD**	<2	_	_	_	_	73/107 (68)	3 (4)
**HFS**	<0.12	_	_	_	_	86/107 (80)	3 (4)

AAR, AST to ALT ratio; ALT, alanine aminotransferase; APRI, AST to Platelet Ratio Index; AST, aspartate aminotransferase; BARD, BMI AST/ALT Ratio Diabetes; BLF, biomarkers of liver fibrosis; CLD, chronic liver disease; FIB-4, fibrosis-4; FN, false negative; HFS, Hepamet fibrosis score; NAFLD, nonalcoholic fatty liver disease; NFS, NAFLD fibrosis score.

**Table 6:** Correlations between liver fibrosis biomarkers and three different cardiovascular risk (CVR) scores.

	SCORE			Progetto CUORE			FRS		
	All	Viral CLD	NAFLD	All	Viral CLD	NAFLD	All	Viral CLD	NAFLD
**AAR**	0.302 *	0.261 ^†^	0.406 *	0.302 *	0.340 *	0.325 ^†^	0.252 *	0.166 ^§^	0.401 *
**APRI**	0.208 *	0.234 ^†^	0.260 *	0.145 ^§^	0.156	0.375 *	0.114	0.124	0.407 *
**FIB-4**	0.462 *	0.476 *	0.499 *	0.402 *	0.399 *	0.560 *	0.342 *	0.319 *	0.584 *
**Forns**	0.468 *	0.454 *	0.544 *	0.437 *	0.370 *	0.568 *	0.445 *	0.453 *	0.532 *
**NFS**	_	_	0.593 *	_	_	0.658 *	_	_	0.702 *
**BARD**	_	_	0.449 *	_	_	0.471 *	_	_	0.540 *
**HFS**	_	_	0.509 *	_	_	0.643 *	_	_	0.679 *

Data are expressed as Pearson’s r correlation coefficients for all correlations except for those with BARD score which are expressed as Spearman’s rho (ρ). * *p* < 0.001 ^†^ *p* < 0.005 ^§^ *p* < 0.05. The absence of any symbols denotes “*p* not significant”. Abbreviations: AAR, AST to ALT ratio; ALT, alanine aminotransferase; APRI, AST to Platelet Ratio Index; AST, aspartate aminotransferase; BARD, BMI AST/ALT Ratio Diabetes; CLD, chronic liver disease; CVR, cardiovascular risk; FIB-4, fibrosis-4; FRS, Framingham risk score; HFS, Hepamet fibrosis score; NAFLD, nonalcoholic fatty liver disease; NFS, NAFLD fibrosis score.

## Data Availability

The data presented in this study are available on request from the corresponding author. The data are not publicly available due to privacy or ethical restrictions.

## Supplementary Materials

The following are available online at https://www.mdpi.com/2075-4418/11/1/98/s1, Tables S1: Characteristics of patients with absent-mild vs. significant liver fibrosis in the whole population, Table S2: Characteristics of patients with absent-mild vs. significant liver fibrosis according to CLD aetiology, Table S3: Characteristics of patients with absent-to-moderate vs. advanced liver fibrosis in the whole population, Table S4: Characteristics of patients with absent-to-moderate vs. advanced liver fibrosis according to CLD etiology, Table S5: Clinical and laboratory characteristics of NAFLD patients with and without NASH, Table S6: Diagnostic performance of liver fibrosis biomarkers for predicting significant liver fibrosis in the whole population (*n* = 280), Table S7: Diagnostic performance of liver fibrosis biomarkers for predicting significant fibrosis in patients with viral CLD (*n* = 173), Table S8: Diagnostic performance of liver fibrosis biomarkers for predicting significant fibrosis in patients with NAFLD (*n* = 107), Figure S1: Diagnostic performance of serum biomarkers for predicting significant fibrosis in all patients, Figure S2: Diagnostic performance of serum biomarkers for predicting significant fibrosis in viral CLD patients, Figure S3: Diagnostic performance of serum biomarkers for predicting significant fibrosis in NAFLD patients.

## Abbreviations

AAR	AST-to-ALT ratio
ALT	alanine aminotransferase
APRI	AST-to platelet ratio index
AST	aspartate aminotransferase
AUROC	area under the receiver operating characteristic
BMI	body mass index
CI	confidence intervals
CVD	cardiovascular disease
CVR	cardiovascular risk
FIB-4	fibrosis-4
FRS	Framingham risk score
GGT	gamma-glutamyltransferase
HBV	hepatitis B virus
HCC	hepatocellular carcinoma
HCV	hepatitis C virus
HDL	high-density lipopoprotein
HFS	hepamet fibrosis score
HOMA-IR	homeostasis model assessment of insulin resistance
IFG	impaired fasting glycemia
LDL	low-density lipopoprotein
NAFLD	nonalcoholic fatty liver disease
NASH	nonalcoholic steatohepatitis
NFS	NAFLD fibrosis score
NPV	negative predictive value
PPV	positive predictive value
ROC	receiver operating characteristic
