# Supplementary material for: Liver Fibrosis Biomarkers Accurately Exclude Advanced Fibrosis and Are Associated with Higher Cardiovascular Risk Scores in Patients with NAFLD or Viral Chronic Liver Disease
Source: Diagnostics (Basel). 2021 Jan 9;11(1):98. doi: 10.3390/diagnostics11010098 (PMC7827076; doi:10.3390/diagnostics11010098)
Supplement: Supplementary file 1 [file diagnostics-11-00098-s001.pdf]

Supplementary material

Liver fibrosis biomarkers accurately exclude advanced fibrosis and are associated with higher cardiovascular risk scores in patients with NAFLD or viral chronic liver disease.

Stefano Ballestri, Alessandro Mantovani, Enrica Baldelli, Simonetta Lugari, Mauro Maurantonio, Fabio Nascimbeni, Alessandra Marrazzo, Dante Romagnoli, Giovanni Targher, Amedeo Lonardo.

Index

A. **Supplementary Tables** **page 2-9**

B. **Supplementary Figures** **page 10-12**

A. Supplementary Tables.

Table S1. Characteristics of patients with absent-mild vs. significant liver fibrosis in the whole population.

Characteristics	Absent-mild fibrosis (n=180)	Significant fibrosis (n=100)	P
Biometrics			
Age (years)	46.2±11.4	50.3±11.4	**0.004**
M/F ratio (n, %)	111/69 (62/38)	74/26 (74/26)	**0.048**
BMI (kg/m^2)	27.0±4.5	28.2±4.4	**0.041**
WC (cm)	95.1±11.7	100.0±12.6	**0.009**
Hypertension (n, %)	47 (26)	35 (35)	0.131
Type 2 diabetes (n, %)	21 (12)	25 (25)	**0.007**
MetS (n, %)	43 (24)	34 (34)	0.093
CVR scores			
SCORE	0.3 (0.1÷1.2)	0.9 (0.2÷1.8)	**<0.001**
FRS	6.4 (3.2÷12.5)	10.1 (4.9÷18.0)	**<0.001**
Progetto CUORE	1.7 (0.7÷4.1)	2.8 (1.1÷6.3)	**0.003**
Laboratory			
Platelet count (x10^3/mm^3)	224.2±56.2	191.8±64.9	**<0.001**
Fasting glucose (mg/dL)	93.0 (85.0÷100.0)	97.5 (92.3÷115.8)	**<0.001**
Fasting insulin (mIU/L)	10.0 (7.1÷14.8)	12.2 (8.1÷17.0)	0.086
HOMA-IR score	2.4 (1.5÷3.7)	3.1 (2.0÷4.8)	**0.017**
AST (U/L)	34.8 (26.4÷51.0)	50.0 (36.3÷70.5)	**<0.001**
ALT (U/L)	56.0 (37.3÷90.8)	91.0 (55.0÷134.2)	**<0.001**
GGT (U/L)	38.8 (22.0÷60.8)	55.0 (36.0÷104.0)	**<0.001**
Albumin (g/dL)	4.4±0.4	4.3±0.5	**0.024**
γ-globulin (mg/dL)	1.2±0.3	1.3±0.5	0.180
TC (mg/dL)	189.6±43.0	181.5±47.7	0.150
HDL-C (mg/dL)	47.2±13.6	45.2±14.4	0.329
LDL-C (mg/dL)	122.4±38.7	113.4±40.3	0.116
TG (mg/dL)	93.0 (71.3÷135.8)	113.0 (68.0÷168.0)	0.158
SUA (mg/dL)	5.2±1.4	5.4±1.5	0.361
Ferritin (mg/dL)	121.5 (62.8÷232.0)	193.0 (111.0÷297.0)	**0.001**
Liver fibrosis biomarkers			
AAR	0.63 (0.51÷0.74)	0.55 (0.46÷0.72)	0.088
APRI	0.50 (0.31÷0.73)	0.71 (0.47÷1.39)	**<0.001**
FIB-4	0.93 (0.69÷1.44)	1.37 (0.84÷2.57)	**<0.001**
Forns	4.4±1.4	5.7±2.1	**<0.001**

Data are expressed as means (±SD) for continuous variables normally distributed or as medians (25th–75th percentile) for those not normally distributed, and as frequencies (percentages) for categorical variables.

Abbreviations: AAR, AST to ALT ratio; ALT, alanine aminotransferase; APRI, AST to Platelet Ratio Index; AST, aspartate aminotransferase; BMI, body mass index; CVR, cardiovascular risk; F, female; FIB-4, fibrosis-4; FRS, Framingham risk score; GGT, gamma-glutamyl transferase; HDL-C, high density lipoprotein cholesterol; HOMA-IR, homeostasis model assessment of insulin resistance; LDL-C, low density lipoprotein cholesterol; M, male; MetS, metabolic syndrome; SUA, serum uric acid; TC, total cholesterol; TG, triglycerides; WC, waist circumference.

Table S2. Characteristics of patients with absent-mild vs. significant liver fibrosis according to CLD aetiology.

	Viral CLD			NAFLD		
**Characteristics**	**Absent-mild fibrosis (n=118)**	**Significant Fibrosis (n=55)**	**P**	**Absent-mild fibrosis (n=62)**	**Significant Fibrosis (n=45)**	**P**
Biometrics						
Age (years)	45.7±11.1	51.8±10.7	**<0.001**	47.0±11.9	48.47±12.1	0.533
M/F ratio (n, %)	70/48 (59/41)	38/17 (69/31)	0.241	41/21 (66/34)	36/9 (80/20)	0.132
BMI (kg/m^2)	25.9±4.2	26.2±3.1	0.646	28.9±4.4	30.4±4.5	0.077
WC (cm)	90.0±10.4	92.7±9.0	0.260	99.5±11.1	104.7±12.4	**0.028**
Hypertension (n, %)	27 (23)	17 (31)	0.261	20 (32)	18 (40)	0.422
Type 2 diabetes (n, %)	6 (5)	7 (13)	0.118	15 (24)	18 (40)	0.093
MetS (n, %)	15 (13)	6 (11)	0.808	28 (45)	28 (62)	0.116
CVR scores						
SCORE	0.3 (0.1÷1.1)	0.8 (0.3÷1.6)	**0.003**	0.5 (0.2÷1.2)	1 (0.2÷2.2)	0.180
FRS	6.2 (3.0÷12.5)	9.2 (4.8÷16.0)	**0.013**	6.9 (3.5÷13.3)	11.4 (5.4÷26.7)	**0.024**
Progetto CUORE	1.3 (0.5÷4.0)	1.9 (0.9÷4.2)	0.119	2 (0.9÷4.2)	4.3 (1.8÷8.8)	**0.018**
Laboratory						
Platelets (x10^3/mm^3)	220.0±56.9	176.4±60.5	**<0.001**	232.0±54.7	210.6±65.7	**0.069**
Glucose (mg/dL)	92.0 (84.0÷99.0)	97.0 (89.0÷104.0)	**0.009**	96.0 (88.0÷105.5)	101.0 (94.0÷123.0)	**0.045**
Insulin (mIU/L)	9.6 (7.1÷15.5)	9.4 (6.7÷16.0)	0.922	10.3 (6.8÷13.1)	14.0 (8.5÷17.9)	**0.024**
HOMA-IR score	2.4 (1.5÷3.6)	2.5 (1.5÷4.4)	0.367	2.5 (1.5÷3.7)	3.4 (2.2÷5.6)	**0.016**
AST (U/L)	39.2 (29.0÷57.5)	61.0 (38.0÷88.0)	**<0.001**	28.0 (24.0÷40.0)	42.0 (29.0÷54.5)	**<0.001**
ALT (U/L)	60.5 (39.8÷105.5)	107.1 (55.5÷154.8)	**0.001**	47.0 (35.0÷71.5)	75.0 (53.5÷109.5)	**0.001**
GGT (U/L)	36.0 (20.0÷48.7)	55.0 (37.0÷89.0)	**<0.001**	48.5 (30.3÷100.5)	54.0 (35.0÷112.5)	0.333
Albumin (g/dL)	4.4±0.4	4.2±0.5	**0.005**	4.5±0.5	4.4±0.4	0.545
γ-globulin (mg/dL)	1.31±0.33	1.50±0.45	**0.003**	1.07±0.21	1.06±0.31	0.987
TC (mg/dL)	175.8±37.0	163.1±37.8	**0.038**	215.7±41.6	204.0±49.2	0.187
HDL-C (mg/dL)	46.9±13.8	50.8±14.7	0.177	47.5±13.5	40.6±12.5	**0.010**
LDL-C (mg/dL)	105.3±30.3	96.9±36.0	0.226	141.8±38.3	126.8±39.1	0.058
TG (mg/dL)	83.0 (62.0÷112.0)	81.0 (61.0÷113.0)	0.773	132.0 (89.0÷206.0)	165.0 (117.5÷238.5)	0.103
SUA (mg/dL)	4.8±1.2	4.8±1.2	0.978	6.0±1.5	6.1±1.5	0.850
Ferritin (mg/dL)	111.5 (57.0÷227.8)	176.0 (84.0÷265.0)	**0.021**	144.5 (83.8÷241.0)	210.5 (121.0÷359.0)	**0.015**
Liver fibrosis biomarkers						
AAR	0.65 (0.53÷0.75)	0.64 (0.50÷0.73)	0.625	0.59 (0.48÷0.69)	0.49 (0.42÷0.68)	0.054
APRI	0.55 (0.35÷0.85)	1.11 (0.60÷1.68)	**<0.001**	0.38 (0.27÷0.54)	0.61 (0.38÷0.81)	**0.001**
Fib4	1.04 (0.74÷1.56)	1.93 (1.05÷3.05)	**<0.001**	0.76 (0.63÷1.09)	1.03 (0.64÷1.73)	0.059
Forns	4.5±1.4	6.3±1.9	**<0.001**	4.2±1.4	5.0±2.1	**0.026**
NFS	_	_	_	-2.05±1.29	-1.35±2.01	**0.045**
BARD	_	_	_	1 (0÷2)	1 (0÷2)	0.208
HFS	_	_	_	0.04 (0.01÷0.07)	0.06 (0.02÷0.20)	**0.026**

Data are expressed as means (±SD) for continuous variables normally distributed or as medians (25th–75th percentile) for those not normally distributed, and as frequencies (percentages) for categorical variables.

Abbreviations: AAR, AST to ALT ratio; ALT, alanine aminotransferase; APRI, AST to Platelet Ratio Index; AST, aspartate aminotransferase; BARD, BMI AAR Diabetes; BMI, body mass index; CVR, cardiovascular risk; F, female; FIB-4, fibrosis-4; FRS, Framingham risk score; GGT, gamma-glutamyl transferase; HDL-C, high density lipoprotein cholesterol; HFS, Hepamet fibrosis score; HOMA-IR, homeostasis model assessment of insulin resistance; LDL-C, low density lipoprotein cholesterol; M, male; MetS, metabolic syndrome; NFS, NAFLD fibrosis score; SUA, serum uric acid; TC, total cholesterol; TG, triglycerides; WC, waist circumference.

Table S3. Characteristics of patients with absent-to-moderate vs. advanced liver fibrosis in the whole population.

Characteristics	Absent to moderate fibrosis (n=242)	Advanced Fibrosis (n=38)	P
Biometrics			
Age (years)	46.3±11.5	56.0±8.0	**<0.001**
M/F ratio (n, %)	159/83 (66/34)	26/12 (68/32)	0.854
BMI (kg/m²)	27.3±4.4	28.7±5.0	0.069
WC (cm)	96.4±11.3	101.4±16.9	0.158
Hypertension (n, %)	63 (26)	19 (50)	**0.004**
Diabetes (n, %)	30 (12)	16 (42)	**<0.001**
MetS (n, %)	63 (26)	14 (38)	0.175
CVR scores			
SCORE	0.4 (0.1÷1.3)	1.3 (0.8÷3.0)	**<0.001**
FRS	6.7 (3.2÷12.9)	15.7 (8.9÷27.8)	**<0.001**
Progetto CUORE	1.8 (0.8÷4.2)	5.7 (2.5÷12.2)	**<0.001**
Laboratory			
Platelets (x10³/mm³)	221.9±55.8	153.2±62.6	**<0.001**
Glucose (mg/dL)	94.0 (86.0÷101.0)	99.0 (92.5÷139.5)	**0.002**
Insulin (mIU/L)	10.6 (7.1÷15.7)	10.7 (7.8÷19.4)	0.516
HOMA-IR score	2.5 (1.6÷3.8)	3.2 (2.0÷6.9)	0.075
AST (U/L)	37.0 (27.7÷53.3)	62.8 (46.5÷82.1)	**<0.001**
ALT (U/L)	61.0 (40.8÷107.3)	93.0 (55.9÷143.3)	**0.004**
GGT (U/L)	40.0 (25.5÷66.5)	71.5 (47.8÷141.3)	**<0.001**
Albumin (g/dL)	4.4±0.4	4.2±0.4	**0.001**
γ-globulin (mg/dL)	1.2±0.4	1.4±0.5	0.086
TC (mg/dL)	189.8±45.2	166.7±36.6	**0.003**
HDL-C (mg/dL)	46.4±14.0	46.3±14.1	0.976
LDL-C (mg/dL)	120.9±39.8	103.6±34.8	**0.049**
TG (mg/dL)	95.0 (69.0÷148.0)	115.0 (75.5÷145.3)	0.402
SUA (mg/dL)	5.3±1.4	5.1±1.4	0.540
Ferritin (mg/dL)	144.5 (75.3÷245.8)	184.0 (92.0÷378.0)	0.070
Liver fibrosis biomarkers			
AAR	0.61 (0.48÷0.72)	0.67 (0.52÷0.85)	**0.042**
APRI	0.51 (0.32÷0.74)	1.26 (0.76÷1.75)	**<0.001**
FIB-4	0.96 (0.69÷1.46)	2.53 (1.73÷3.59)	**<0.001**
Forns	4.5±1.5	7.3±1.6	**<0.001**

Data are expressed as means (±SD) for continuous variables normally distributed or as medians (25th–75th percentile) for those not normally distributed, and as frequencies (percentages) for categorical variables.

Abbreviations: AAR, AST to ALT ratio; ALT, alanine aminotransferase; APRI, AST to Platelet Ratio Index; AST, aspartate aminotransferase; BMI, body mass index; CVR, cardiovascular risk; F, female; FIB-4, fibrosis-4; FRS, Framingham risk score; GGT, gamma-glutamyl transferase; HDL-C, high density lipoprotein cholesterol; HOMA-IR, homeostasis model assessment of insulin resistance; LDL-C, low density lipoprotein cholesterol; M, male; MetS, metabolic syndrome; SUA, serum uric acid; TC, total cholesterol; TG, triglycerides; WC, waist circumference.

Table S4. Characteristics of patients with absent-to-moderate vs. advanced liver fibrosis according to CLD etiology.

	Viral CLD (n=173)			NAFLD (n=107)		
**Characteristics**	**Absent-to-moderate fibrosis (n=149)**	**Advanced fibrosis (n=24)**	**P**	**Absent-to-moderate fibrosis (n=93)**	**Advanced fibrosis (n=14)**	**P**
Biometrics						
Age (years)	46.5±11.4	54.8±7.6	**<0.001**	46.1±11.6	58.0±8.5	**<0.001**
Sex M/F (n, %)	93/56 (62/38)	15/9 (63/37)	1.000	66/27 (71/29)	11/3 (79/21)	0.753
WC (cm)	91.2±9.9	89.9±10.5	0.688	100.3±10.7	111.1±15.2	**0.021**
Hypertension (n, %)	35 (24)	9 (37)	0.205	28 (30)	10 (71)	**0.005**
BMI (kg/m^2)	25.9±3.9	26.6±3.9	0.452	29.1±4.4	32.0±4.9	**0.025**
Type 2 diabetes (n, %)	9 (6)	4 (17)	0.086	21 (23)	12 (86)	**<0.001**
MetS (n, %)	18 (12)	3 (12)	1.000	45 (48)	11 (79)	**0.045**
CVR scores						
SCORE	0.3 (0.6÷1.2)	1.0 (0.5÷2.8)	**0.002**	0.4 (0.1÷1.4)	2.2 (1.2÷3.3)	**<0.001**
FRS	6.4 (3.2÷12.6)	11.4 (5.0÷18.0)	**0.009**	7.3 (3.4÷15.0)	27.8 (14.7÷45.5)	**<0.001**
Progetto CUORE	1.5 (0.5÷4.0)	2.6 (1.0÷5.7)	0.152	2.0 (0.9÷4.5)	9.4 (5.1÷17.7)	**<0.001**
Laboratory						
Platelets (x10^3/mm^3)	216.1±56.3	144.8±56.5	**<0.001**	231.3±53.9	167.6±71.8	**<0.001**
Glucose (mg/dL)	93.0 (84.0÷100.0)	94.0 (88.0÷112.0)	0.242	96.0 (89.0÷108.5)	132.0 (101.8÷191.8)	**<0.001**
Insulin (mIU/L)	9.7 (7.1÷15.9)	8.7 (6.8÷14.9)	0.719	11.5 (7.8÷15.4)	13.7 (8.7÷27.1)	0.150
HOMA-IR score	2.4 (1.5÷3.6)	2.3 (1.5÷4.8)	0.632	2.7 (1.7÷4.1)	4.1 (2.1÷12.2)	**0.024**
AST (U/L)	40.5 (30.5÷60.0)	73.1 (55.0÷93.3)	**<0.001**	31.0 (25.0÷42.0)	50.2 (45.0÷56.3)	**<0.001**
ALT (U/L)	64.0 (42.0÷123.5)	124.5 (75.8÷166.5)	**0.006**	58.0 (39.5÷94.0)	75.0 (44.4÷92.0)	0.224
GGT (U/L)	37.0 (22.5÷54.0)	72.4 (42.3÷136.3)	**<0.001**	48.0 (31.0÷96.5)	71.5 (47.8÷154.0)	**0.023**
Albumin (g/dL)	4.4±0.4	4.2±0.4	**0.015**	4.5±0.43	4.2±0.52	**0.030**
γ-globulin (mg/dL)	1.34±0.37	1.55±0.44	**0.014**	1.06±0.24	1.09±0.37	0.816
TC (mg/dL)	173.9±38.9	158.9±25.5	**0.019**	215.4±42.9	180.1±48.6	**0.006**
HDL-C (mg/dL)	47.4±14.1	55.8±12.6	0.061	45.3±13.8	38.9±10.5	0.096
LDL-C (mg/dL)	103.0±32.7	96.0±31.8	0.519	139.1±38.2	109.6±37.1	**0.010**
TG (mg/dL)	81.0 (62.0÷109.5)	93.0 (63.0÷123.0)	0.356	145.0 (89.0÷234.3)	146.0 (119.0÷215.0)	0.815
SUA (mg/dL)	4.8±1.2	4.9±1.4	0.778	6.1±1.5	5.5±1.4	0.182
Ferritin (mg/dL)	122.5 (62.0÷232.0)	184.0 (114.0÷384.0)	0.065	178.0(100.8÷247.8)	193.0 (85.8÷392.5)	0.561
Liver fibrosis biomarkers						
AST/ALT AAR	0.64 (0.51÷0.74)	0.68 (0.55÷0.76)	0.357	0.56 (0.45÷0.67)	0.59 (0.51÷0.99)	0.054
APRI	0.59 (0.39÷0.89)	1.52 (1.17÷1.88)	**<0.001**	0.41 (0.29÷0.63)	0.85 (0.65÷1.20)	**<0.001**
Fib4	1.07 (0.77÷1.60)	2.58 (2.13÷3.54)	**<0.001**	0.74 (0.61÷1.10)	2.19 (1.31÷3.73)	**<0.001**
Forns	4.7±1.5	7.5±1.6	**<0.001**	4.1±1.4	7.1±1.8	**<0.001**
NFS	_	_	_	-2.08±1.40	0.43±1.62	**<0.001**
BARD	_	_	_	1 (0÷1)	2 (2÷4)	**0.001**
HFS	_	_	_	0.04 (0.01÷0.07)	0.34 (0.19÷0.70)	**<0.001**

Data are expressed as means (±SD) for continuous variables normally distributed or as medians (25th–75th percentile) for those not normally distributed, and as frequencies (percentages) for categorical variables.

Abbreviations: AAR, AST to ALT ratio; ALT, alanine aminotransferase; APRI, AST to Platelet Ratio Index; AST, aspartate aminotransferase; BARD, BMI AAR Diabetes; BMI, body mass index; CVR, cardiovascular risk; F, female; FIB-4, fibrosis-4; FRS, Framingham risk score; GGT, gamma-glutamyl transferase; HDL-C, high density lipoprotein cholesterol; HFS, Hepamet fibrosis score; HOMA-IR, homeostasis model assessment of insulin resistance; LDL-C, low density lipoprotein cholesterol; M, male; MetS, metabolic syndrome; NFS, NAFLD fibrosis score; SUA, serum uric acid; TC, total cholesterol; TG, triglycerides; WC, waist circumference.

Table S5. Characteristics of patients with simple steatosis vs. NASH in the NAFLD population.

Characteristics	Simple steatosis (n=30)	NASH (n=77)	P
Biometrics			
Age (years)	46.2±12.5	48.2±11.8	0.446
M/F ratio (n, %)	21/9 (70/30)	56/21 (73/27)	0.813
BMI (kg/m^2)	26.9±3.5	30.5±4.5	**<0.001**
WC (cm)	93.8±8.0	104.7±11.8	**<0.001**
Hypertension (n, %)	6 (20)	32 (42)	**0.044**
Type 2 diabetes (n, %)	7 (23)	26 (34)	0.356
MetS (n, %)	8 (27)	48 (62)	**0.001**
CVR scores			
SCORE	0.6 (0.1÷1.3)	0.7 (0.2÷1.9)	0.433
FRS	6.5 (3.1÷12.1)	10.4 (4.1÷23.7)	0.057
Progetto CUORE	1.8 (0.8÷3.6)	3.5 (1.1÷7.0)	**0.016**
Laboratory			
Platelet count (x10^3/mm^3)	230.0±50.5	220.3±63.7	0.453
Fasting glucose (mg/dL)	95.5 (87.8÷110.5)	98.0 (91.5÷119.0)	0.179
Fasting insulin (mIU/L)	7.3 (5.2÷10.6)	13.0 (9.5÷17.8)	**<0.001**
HOMA-IR score	1.7 (1.3÷2.4)	3.4 (2.3÷4.7)	**<0.001**
AST (U/L)	27.9 (20.8÷35.3)	38.0 (27.0÷53.0)	**0.002**
ALT (U/L)	41.0 (35.0÷63.3)	65.0 (45.0÷106.0)	**0.002**
GGT (U/L)	48.0 (35.5÷101.3)	51.0 (31.5÷110.0)	0.920
Albumin (g/dL)	4.6±0.4	4.4±0.5	0.206
γ-globulin (mg/dL)	1.1±0.3	1.1±0.3	0.962
TC (mg/dL)	221.0±45.3	206.8±44.6	0.145
HDL-C (mg/dL)	50.7±14.1	42.1±12.5	**0.004**
LDL-C (mg/dL)	142.8±38.5	132.4±39.2	0.243
TG (mg/dL)	107.0 (78.8÷189.8)	153.0 (115.0÷237.0)	**0.035**
SUA (mg/dL)	5.4±1.4	6.3±1.4	**0.004**
Ferritin (mg/dL)	132.0 (86.0÷226.0)	195.0 (102.7÷319.5)	**0.062**
Liver histology			
Significant fibrosis (n, %)	2 (7)	43 (56)	**<0.001**
Advanced fibrosis (n, %)	0 (0)	14 (18)	**0.010**
Cirrhosis (n, %)	0 (0)	5 (6)	0.319
Liver fibrosis biomarkers			
AAR	0.59 (0.51÷0.70)	0.51 (0.43÷0.68)	0.106
APRI	0.36 (0.23÷0.48)	0.51 (0.34÷0.72)	**0.005**
FIB-4	0.73 (0.58÷1.1)	0.91 (0.66÷1.42)	0.091
Forns	4.1±1.6	4.7±1.8	0.140
NFS	-2.30±1.37	-1.54±1.72	**0.033**
BARD	1 (0÷1)	1 (1÷2)	**0.018**
HFS	0.02 (0.01÷0.07)	0.05 (0.02÷0.16)	**0.008**

Data are expressed as means (±SD) for continuous variables normally distributed or as medians (25th–75th percentile) for those not normally distributed, and as frequencies (percentages) for categorical variables.

Abbreviations: AAR, AST to ALT ratio; ALT, alanine aminotransferase; APRI, AST to Platelet Ratio Index; AST, aspartate aminotransferase; BARD, BMI AAR Diabetes; BMI, body mass index; CVR, cardiovascular risk; F, female; FIB-4, fibrosis-4; FRS, Framingham risk score; GGT, gamma-glutamyl transferase; HDL-C, high density lipoprotein cholesterol; HFS, Hepamet fibrosis score; HOMA-IR, homeostasis model assessment of insulin resistance; LDL-C, low density lipoprotein cholesterol; M, male; MetS, metabolic syndrome; NFS, NAFLD fibrosis score; NASH, nonalcoholic steatohepatitis; SUA, serum uric acid; TC, total cholesterol; TG, triglycerides; WC, waist circumference.

Table S6. Diagnostic performance of liver fibrosis biomarkers for predicting significant liver fibrosis in the whole population (n=280).

	Cut-offs	SE	SP	PPV	NPV	ACC	LR+	LR-	AUROC (95%CI)
**AAR**	≥ 0.8	15.0	81.7	31.3	63.4	57.9	0.82	1.04	0.44 (0.40-0.51)
	> 1	9.0	93.9	45.0	65.0	63.6	1.47	0.97	
**APRI**	> 0.5	72.0	51.7	45.3	76.9	58.9	1.49	0.54	0.68 (0.62- 0.75)
	> 0.7	50.0	70.6	48.5	71.8	63.2	1.70	0.71	
	> 1.5	19.0	94.4	65.5	67.7	67.5	3.42	0.86	
**Fib-4**	≥ 1.45	48.0	75.0	51.6	72.2	65.4	1.92	0.69	0.66 (0.59-0.73)
	> 3.25	18.0	97.8	81.8	68.2	69.3	8.10	0.84	
**Forns**	≥ 4.2	78.0	47.2	45.1	79.4	58.2	1.48	0.47	0.69 (0.63-0.76)
	> 6.9	29.0	95.6	78.4	70.8	71.8	6.53	0.74	

Significant fibrosis was defined as histologic liver fibrosis ≥F3 according to Ishak et al. [29] for viral CLD and ≥F2 according to Brunt/Kleiner et al. for NAFLD [27, 28].

Abbreviations: AAR, AST to ALT ratio; ACC, accuracy; ALT, alanine aminotransferase; APRI, AST to Platelet Ratio Index; AST, aspartate aminotransferase; AUROC, area under the receiver operating characteristics; CLD, chronic liver disease; Fib-4, fibrosis 4; LR, likelihood ratio; NPV, negative predictive value; PPV, positive predictive value; SE, sensitivity; SP, specificity.

Table S7. Diagnostic performance of liver fibrosis biomarkers for predicting significant fibrosis in patients with viral CLD (n=173).

	Cut-offs	SE	SP	PPV	NPV	ACC	LR+	LR-	AUROC (95%CI)
**AAR**	≥ 0.8	14.6	79.7	25.0	66.7	59.0	0.72	1.07	0.48 (0.38-0.57)
	> 1	9.1	93.2	38.5	68.8	66.5	1.34	0.98	
**APRI**	> 0.5	83.6	42.4	40.4	84.8	55.5	1.45	0.39	0.73 (0.65-0.81)
	> 0.7	63.6	61.9	43.8	78.5	62.4	1.67	0.59	
	> 1.5	30.9	92.4	65.4	74.3	72.8	4.05	0.75	
**FIB-4**	≥1.45	60.0	68.6	47.1	78.6	65.9	1.91	0.58	0.73 (0.65-0.81)
	> 3.25	21.8	97.5	80.0	72.8	73.4	8.58	0.80	
**Forns**	≥ 4.2	89.1	42.4	41.9	89.3	57.2	1.55	0.26	0.77 (0.69-0.85)
	> 6.9	40.0	95.8	81.5	77.4	78.0	9.44	0.63	

Significant fibrosis was defined as histologic liver fibrosis ≥F3 according to Ishak et al. [29].

Abbreviations: AAR, AST to ALT ratio; ACC, accuracy; ALT, alanine aminotransferase; AST, aspartate aminotransferase; APRI, ALT to Platelet Ratio Index; AUROC, area under the receiver operating characteristics; CLD, chronic liver disease; FIB-4, fibrosis 4; LR, likelihood ratio; NPV, negative predictive value; PPV, positive predictive value; SE, sensitivity; SP, specificity.

Table S8. Diagnostic performance of liver fibrosis biomarkers for predicting significant fibrosis in patients with NAFLD (n=107).

	Cut-offs	SE	SP	PPV	NPV	ACC	LR+	LR-	AUROC (95% CI)
**AAR**	≥ 0.8	15.6	85.5	43.8	58.2	56.1	1.07	0.99	0.39 (0.28-0.51)
	> 1	8.9	95.2	57.1	59.0	58.9	1.84	0.96	
**APRI**	> 0.5	57.8	69.4	57.8	69.4	64.5	1.89	0.61	0.69 (0.59-0.80)
	> 0.7	33.3	87.1	65.2	64.3	64.5	2.58	0.77	
	> 1.5	4.4	98.4	66.7	58.7	58.9	2.76	0.97	
**FIB-4**	≥ 1.3	37.8	85.5	65.4	65.4	65.4	2.60	0.73	0.61 (0.49-0.72)
	> 2.67	13.3	96.8	75.0	60.6	61.7	4.13	0.90	
	≥1.45	33.3	87.1	65.2	64.3	64.5	2.58	0.77	
	> 3.25	13.3	98.4	85.7	61.0	62.6	8.27	0.88	
**Forns**	≥ 4.2	64.4	56.5	51.8	68.6	59.8	1.48	0.63	0.62 (0.52-0.73)
	> 6.9	15.6	95.2	70.0	60.8	61.7	3.21	0.89	
**NFS**	≥ -1.455	53.3	66.1	53.3	66.1	60.8	1.58	0.71	0.61 (0.50-0.73)
	> 0.675	15.6	96.8	77.8	61.2	62.6	4.82	0.87	
**BARD**	≥ 2	37.8	72.6	50.0	61.6	57.9	1.38	0.86	0.56 (0.45-0.67)
**HFS**	≥ 0.12	31.1	88.7	66.7	64.0	64.5	2.76	0.78	0.63 (0.52-0.74)
	> 0.47	20.0	98.4	90.0	62.9	65.4	12.4	0.81	

Significant fibrosis was defined as histologic liver fibrosis ≥F2 according to Brunt/Kleiner et al. [27, 28].

Abbreviations: AAR, AST to ALT ratio; ACC, accuracy; ALT, alanine aminotransferase; AST, aspartate aminotransferase; APRI, ALT to Platelet Ratio Index; AUROC, area under the receiver operating characteristics; BARD, BMI AAR Diabetes; CLD, chronic liver disease; FIB-4, fibrosis-4; HFS, Hepamet fibrosis score; LR, likelihood ratio; NFS, NAFLD fibrosis score; NPV, negative predictive value; PPV, positive predictive value; SE, sensitivity; SP, specificity.

B. Supplementary Figures.

Figure S1. Diagnostic performance of serum biomarkers for predicting significant fibrosis in all patients.

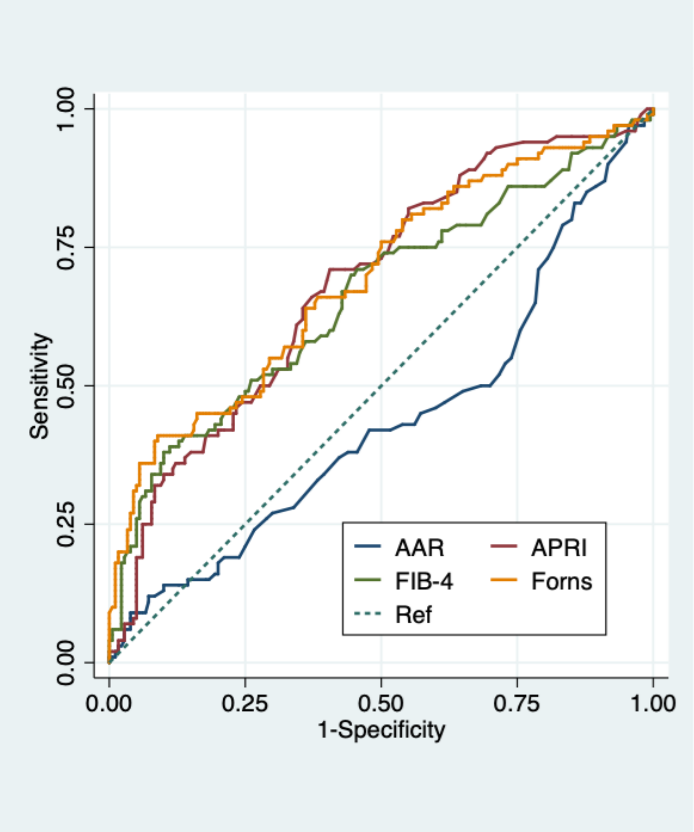

Receiver operating characteristic (ROC) curves of serum biomarkers for the diagnosis of significant fibrosis in all patients. AUROC (95% CI): 0.44 (0.40-0.51) for AAR, 0.68 (0.62- 0.75) for APRI, 0.66 (0.59-0.73) for Fib-4, 0.69 (0.63-0.76) for Forns index. Significant fibrosis was defined as histologic liver fibrosis ≥F3 according to Ishak et al. [29] for viral CLD, and ≥F2 according to Brunt/Kleiner et al. for NAFLD [27, 28].

AAR, AST to ALT ratio; APRI, AST to Platelet Ratio Index; Fib-4, fibrosis 4.

Figure S2. Diagnostic performance of serum biomarkers for predicting significant fibrosis in viral CLD patients.

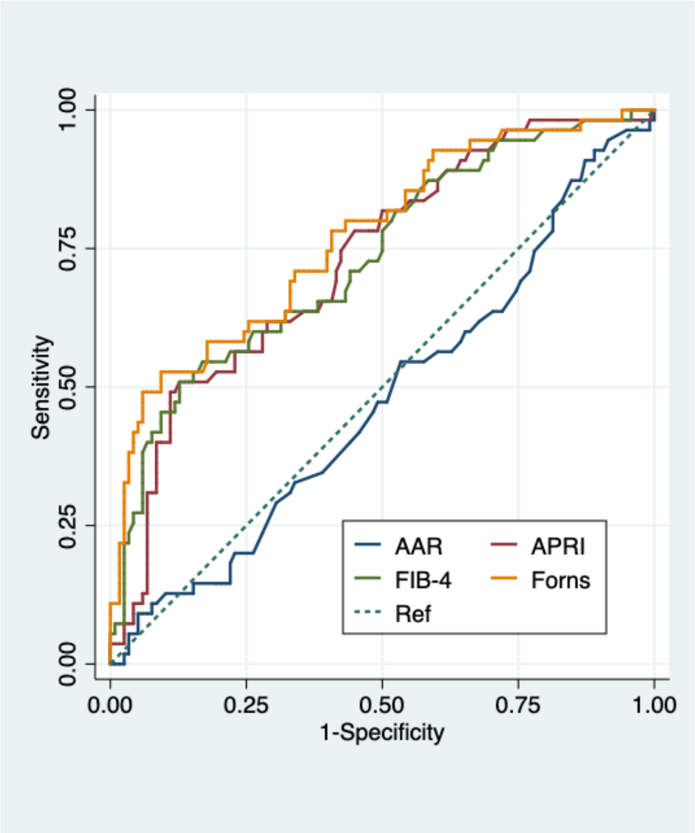

ROC curves of serum biomarkers for the diagnosis of significant fibrosis in viral CLD patients. AUROC (95% CI): 0.48 (0.38-0.57) for AAR, 0.73 (0.65-0.81) for APRI, 0.73 (0.65-0.81) for FIB-4, 0.77 (0.69-0.85) for Forns index. Significant fibrosis was defined as histologic liver fibrosis ≥F3 according to Ishak et al. [29].

AAR, AST to ALT ratio; APRI, AST to Platelet Ratio Index; Fib-4, fibrosis 4.

Figure S3. Diagnostic performance of serum biomarkers for predicting significant fibrosis in NAFLD patients.

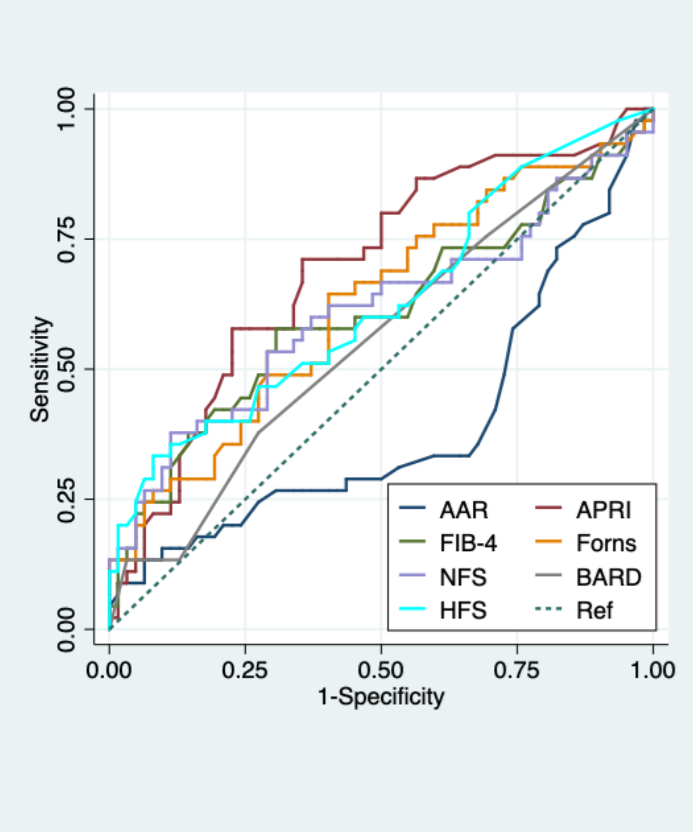

ROC curves of serum biomarkers for the diagnosis of significant fibrosis in NAFLD patients. AUROC (95% CI): 0.39 (0.28-0.51) for AAR, 0.69 (0.59-0.80) for APRI, 0.61 (0.49-0.72) for FIB-4, 0.62 (0.52-0.73) for Forns index, 0.61 (0.50-0.73) for NFS, 0.63 (0.52-0.74) for HFS, 0.56 (0.45-0.67) for BARD. Advanced fibrosis was defined as histologic liver fibrosis ≥F4 according to Ishak et al. [29] for viral CLD, and ≥F3 according to Brunt/Kleiner et al. for NAFLD [27, 28].

AAR, AST to ALT ratio; APRI, AST to Platelet Ratio Index; BARD, BMI AAR Diabetes; Fib-4, fibrosis 4; HFS, Hepamet fibrosis score; NFS, NAFLD fibrosis score.
